# Fetal Alcohol Spectrum Disorders: Fixing Our Aim to Aim for the Fix

**DOI:** 10.3390/ijerph16203978

**Published:** 2019-10-18

**Authors:** Larry Burd, Svetlana Popova

**Affiliations:** 1 North Dakota Fetal Alcohol Syndrome Center, Department of Pediatrics, University of North Dakota School of Medicine and Health Sciences, Pediatric Therapy Services, Altru Health System, Grand Forks, ND 58202, USA; 2 Institute for Mental Health Policy Research & Campbell Family Mental Health Research Institute, Centre for Addiction and Mental Health, (CAMH), Toronto, ON M6J 1H4, Canada; Lana.Popova@camh.ca; 3 Epidemiology Division, Office of Global Public Health Education & Training, Dalla Lana School of Public Health, University of Toronto, Toronto, ON M5S 1A1, Canada; 4 Factor-Inwentash Faculty of Social Work, University of Toronto, Toronto, ON M5S 1A1, Canada

We, as editors of this special issue on Fetal Alcohol Spectrum Disorders (FASD), are proud to present eleven papers [1,2,3,4,5,6,7,8,9,10,11]. These studies focused on prevention, systems development, clinical practice, and public policy. These are most welcome enhancements to the scientific evidence base on FASD. 

FASD continues to be an enigma. Prevalence rates are high, but rates of diagnosis are low. FASD poses a huge clinical and public health burden, yet it receives only modest attention from clinicians. FASD is more prevalent than autism and providing care and interventions for individuals with FASD is more expensive. Yet these services receive only a small fraction of what is allocated for autism. FASD is potentially preventable but funding for prevention is also minimal. FASD imposes a huge burden on mental health, child welfare, and correctional systems. Yet, equivalent investments in research or clinical practice are lacking. This has been the state of FASD for decades. 

In the Factsheet, presented below, we summarize the current state of FASD. We finally need to face the facts! FASD is a common disorder, which is rarely diagnosed and, as a result, nearly always treated as something else. This has consequences that are devastating for people with FASD and their families. 

Four common issues are at the root of this problem. 

Firstly, the belief that FASD must be diagnosed by a multidisciplinary team. FASD is common; there are 1726 to 17,810 new cases every DAY globally. Currently, we cannot diagnose even 1% of these cases. There are 11.3 MILLION affected people 18 years old or younger. Even a moment of reflection should startle us to the realization that we do not have the capacity to provide multidisciplinary evaluations for a population of this size. Even the wealthiest countries cannot provide this service. The good news is, it is not needed. Most individuals with FASD are in systems of care that with a few modest modifications can greatly improve care for people with FASD and their families.

Secondly, we now have to face the facts—in FASD—it is not the face. Most of the complexity of FASD is due to other problems. Prematurity, growth impairments, brain damage, victimization, and the inability to avoid exploitation and to live independently are the central problems. 

Thirdly, Fetal Alcohol Syndrome (FAS)—the most severe and visible form of FASD is rarely diagnosed at delivery, in the neonatal intensive care nursery or during well childcare. Obstetricians, midwives, labor and delivery teams, and neonatal intensive care teams have demonstrated the ability to respond to prenatal substance exposure consequent to the opioid crisis. We need to see similar changes to improve care for infants and families impacted by prenatal alcohol exposure. 

Fourthly, pediatricians, family doctors, early intervention teams, social workers, the judiciary, and mental health providers see children with FASD. We need a clinical diagnosis that can be implemented in these settings. The phenotype of FASD is expressed in these systems. Currently, care provided by schools, psychiatrists, developmental medicine, residential care facilities, and foster care systems, are too often followed by juvenile corrections, more mental health concerns, and substance use. 

In contrast, FASD should be a clinical story about early intervention to prevent recurrence, interventions to prevent exposure to adverse childhood experiences (ACEs), services before, during and after foster care placements, and intensive intervention before FASD severity requires residential care and involvement with juvenile corrections. 

While we do have severe problems with care provided for children and adolescents with FASD, we have far more serious problems with identification and care for adults and the elderly with FASD. Our current policies reflect the view that no one with FASD becomes elderly. In the United States and Canada alone, there are millions of undiagnosed adults and elderly people with FASD.

Existing care pathways for severe FASD, which involves growth impairments, congenital anomalies, and abnormalities of the face, are already integrated across the continuum of care in the United States and Canada. We just need to identify the etiology. It is the common and typical manifestation of FASD that we are missing. It is brain damage, exposure to early and often severe adversity, and a lack of anticipatory intervention that are our current problems. FASD is a disorder of development—a developmental disability with onset at birth. It is a mental health disorder with high rates of comorbidity, which affects parents, caretakers, schools, juvenile corrections, residential care systems, and society. In FASD, diagnosis-informed care should be our mantra. We need to accept that it is common, it is costly, it is often severe, and it is rarely diagnosed. As a result, people with FASD are often misdiagnosed and provided suboptimal treatment.

What is the solution?

- Develop universal clinical diagnostic guidelines for clinicians who care for people with FASD. It must be brief and composed of the clinical language that these clinicians use in their day-to-day practice. It must be included in diagnostic nomenclatures and it must be a reimbursable diagnosis (a diagnosis with billing codes). The diagnosis must enhance our capacity for early diagnosis before exposure to ACEs and the avalanche of secondary disabilities, which exacerbate the overall severity of FASD and increase both the burden and cost of care. Having a diagnosis of FASD is a protective factor.
- Undergraduate and postgraduate medical education and other health care provider curriculum, assessment, and accreditation must include the risks of prenatal alcohol use and FASD. Also, we need to obligate medical doctors, family physicians, and obstetricians to ask women of childbearing age and pregnant women about their alcohol use in order to detect women whose children are at risk for FASD. This should lead to universal screening for alcohol use and provision of brief interventions and referral to treatment, where appropriate, to all pregnant women and women of childbearing age.
- We should provide postpartum support for new mothers, especially mothers of children with FASD, in order to prevent intergenerational re-occurrence of FASD.
- Affected people and their families need to access to timely and diagnosis-informed interventions and ongoing support to people with FASD and their families. We need to develop surveillance systems for FASD and prenatal alcohol exposure in order to monitor trends in incidence and prevalence and to evaluate the effectiveness of our initiatives and interventions.
- We need to better educate our population including children and adolescents (both girls and boys, women and men) about the detrimental consequences of alcohol use during pregnancy. We must inform all women of childbearing age that they should abstain from any type of alcohol use during their entire pregnancy and even when they are trying to get pregnant.
- Finally, our target should be zero alcohol consumption during pregnancy and thus, zero new FASD cases. Meeting this target is possible, if we act now. The WHO’s Global strategy to reduce the harmful use of alcohol [12] and the WHO’s Global action plan for the prevention and control of non-communicable diseases 2013–2020 [13] highlight ten policy areas for multi-sectoral national action to protect the health of populations and reduce the alcohol-attributable disease burden, including FASD.

To conclude, in FASD, the facts provide a face for the disorder and it rarely includes the face (Table 1). The phenotype is mostly behind the face. FASD is mostly a disorder of brain damage and the developmental consequences of brain dysfunction that result in impairments. 

So, let us face it, we need change. 

## Figures and Tables

**Table 1:** Fetal alcohol spectrum disorders (FASD): A 2019 Fact Sheet.

Prevalence of Alcohol Use in Pregnancy		Reference
Early pregnancy alcohol use (USA)	50%	[14]
Alcohol use at end of pregnancy (USA)	1%–7%	[14]
Alcohol use at any time of pregnancy (globally)	10%	[15]
Binge drinking during pregnancy (globally)	10.7% to 31.0% (in Europe and Africa, respectively)	[16]
Women drinking during pregnancy identified by prenatal care providers (USA)	1%–3%	[17]
Women consuming alcohol on the same day they give birth (USA)	34,285	[18]
Alcohol withdrawal in newborn (globally)	Rarely recognized; only 1 case reported every 10 years	[18]
**Neonatal Risks Associated with Prenatal Alcohol Exposure (PAE)**		
Developing FASD among exposed children	1 in 13	[19]
Risk for stillbirth	40% higher	[20]
PAE at the end of pregnancy and risk of SIDS for siblings of child with FASD	10.2× higher	[21]
PAE at the end of pregnancy and risk of death from infectious diseases for siblings of child with FASD	13.7× higher	[21]
Mortality risk for sibling of a child with FAS	6.3× higher	[22]
Risk of recurrence in families with one affected child (FASD)	77% higher	[23]
**Fetal Alcohol Spectrum Disorder (FASD)**		
**Prevalence and Incidence Estimates**		
Newborns identified in NICU with FASD each year (USA)	<70 per year	[19]
New FASD cases per day (globally)	1726	[24]
New FASD cases per day (globally)	1% prevalence = 3,562	[19]
	5% prevalence = 17,810	[25]
Global FASD population: Children/youth (birth–18 years)	11,339,820	See calculations *1
Global FASD population: Adults with FASD (19–64 years)	28,349,550	See calculations *2
Global FASD population: Elderly with FASD (65+)	4,409,930	See calculations *3
FASD prevalence/10,000 (globally)	62.7	Calculated based on Reference [16]
**FASD and Health and Mental Health Impairments**		
Number of comorbid disease conditions associated with FASD (globally)	Over 400	[26]
Risk of child or adolescent psychosis	24.5× higher	[27]
Risk of intellectual disability	23× higher	[27]
Risk of ADHD	10× higher	[27]
Risk of anxiety disorder	11× higher	[27]
Risk of FASD if ACEs score: 2–6	2.1× higher	[28]
Risk of FASD if ACEs score: 7–10	8.1× higher	[28]
**Economic Cost**		
Annual cost of care per child (globally)	$22,810	[29]
Annual cost of care per adult (globally)	$24,308	[29]
Lifetime cost of care per diagnosed individual by age 43	Over $1 million	Calculated based on *4
**Special Sub-Populations**		
Children/youth with FASD in foster care placement	32%–40% higher than in general population	[30]
Children/youth with FASD in special education	10× higher than in general population	[30]
Children/youth with FASD in Canadian juvenile corrections	19× higher risk of incarceration	[31]
Diagnosed with FASD in adult prison	0.0009%	[32]
Undiagnosed/misdiagnosed in adult prison	99.9%	[32]
**Mortality Rates**		
Individuals with FASD	4.3× higher	Calculated based on References [21,22]
Siblings of individuals with FASD	5.3× higher	[21]
Birth mother of individual with FASD	44.8× higher	[33]
**Global Diagnostic Demand and Capacity**		
FASD diagnostic demand	14,560/day	[19]
FASD diagnostic capacity: NICU	30/day	[19]
FASD diagnostic capacity: Children/youth	200–400/day	[19]
FASD diagnostic capacity: Adults	100/day	[19]
FASD diagnostic capacity: Elderly	10/day	Calculated based on Reference [19]
FASD diagnostic capacity: Juvenile corrections	30–50/day	[19]
FASD diagnostic capacity: Adult corrections	30/day	[19]
% of people with FASD diagnosed each year	<1%	Calculated based on Reference [19]
Diagnostic capacity using current FAS phenotype	Only 2% of FASD	Calculated based on Reference [19]

ACEs: adverse childhood experiences; ADHD: attention deficit hyperactivity disorder; FAS: fetal alcohol syndrome; NICU: neonatal intensive care unit; SIDS: sudden infant death syndrome. * 1. New cases per year [16,19] = 1726 per day × 365 = 629,990 cases per year × 18 years = 11,339,820. * 2. New cases per year [16,19] = 1726 per day × 365 = 629,990 cases per year × 45 years = 28,349,550. * 3. New cases per year [16,19] = 1726 per day × 365 = 629,990 cases per year × 7 years (from 65 to 71 years) = 4,409,930. * 4. Annual cost of care for children and youth = $22,810 (birth through 20) and cost for adults = $24,308 (from 21 and up) [19]; ($22,810 × 20 years) + ($24,308 × 23 years) = $1,015,284.
